# Study on the Aging Precipitation Behavior and Kinetics of Al-10.0Zn-3.0Mg-2.8Cu Alloy by Pre-Deformation Treatment

**DOI:** 10.3390/ma17153729

**Published:** 2024-07-27

**Authors:** Zhaolong Fu, Xi Zhao, Minhang Jiao, Xianwei Ren, Hongbin Liu, Hailong Liu

**Affiliations:** 1 Beijing Xinghang Electromechanical Equipment Co., Ltd., Beijing 100074, China; hyy798646088@163.com; 2 School of Aerospace Engineering, North University of China, Taiyuan 030051, China; j1658782773@163.com; 3 School of Materials Science and Engineering, North University of China, Taiyuan 030051, China; 20210028@nuc.edu.cn; 4 Shandong Zhuoyue Aluminum Group Co., Ltd., Jining 272000, China; xhy9801@163.com (H.L.); w18295921095@163.com (H.L.)

**Keywords:** Al-Zn-Mg-Cu alloy, large plastic deformation, precipitation phase, strengthening mechanism, precipitation kinetics

## Abstract

In this paper, the effect of thermomechanical treatment process on the hardening behavior, grain microstructure, precipitated phase, and tensile mechanical properties of the new high-strength and high-ductility Al-10.0Zn-3.0Mg-2.8Cu alloy was studied, and the optimal thermomechanical treatment process was established. The strengthening and toughening mechanisms were revealed, which provided technical and theoretical guidance for the engineering application of this kind of high strength-ductility aluminum alloy. Al-10.0Zn-3.0Mg-2.8Cu alloy cylindrical parts with external longitudinal reinforcement were prepared by a composite extrusion deformation process (reciprocal upsetting + counter-extrusion) with a true strain up to 2.56, and the organizational evolution of the alloys during the extrusion deformation process and the influence of pre-stretching treatments on the subsequent aging precipitation behaviors and mechanical properties were investigated. The results show that firstly, the large plastic deformation promotes the fragmentation of coarse insoluble phases and the occurrence of dynamic recrystallization, which results in the elongation of the grains along the extrusion direction, and the volume fraction of recrystallization reaches 42.4%. Secondly, the kinetic study showed that the decrease in the activation energy of precipitation increased the nucleation sites, which further promoted the diffuse distribution of the second phase in the alloy and a higher number of nucleation sites, while limiting the coarsening of the precipitated phase. When the amount of pre-deformation was increased from 0% to 2%, the size of the matrix precipitated phase decreased from 5.11 μm to 4.1 μm, and when the amount of pre-deformation was increased from 2% to 7%, the coarsening of the matrix precipitated phase took place, and the size of the phase increased from 4.1 μm to 7.24 μm. The finalized heat treatment process for the deformation of the aluminum alloy tailframe was as follows: solution (475 °C/3 h) + 2% pre-stretching + aging (120 °C/24 h), at which the comprehensive performance of the alloy was optimized, with a tensile strength of 634.2 MPa, a yield strength of 571.0 MPa, and an elongation of 15.2%. The alloy was strengthened by both precipitation strengthening and dislocation strengthening. After 2% pre-stretching, the fracture surface starts to be dominated by dense tough nest structure, and most of them are small tough nests, and small and dense tough nests are the main reason for the increase in alloy toughness after 2% pre-stretching deformation.

## 1. Introduction

Al-Zn-Mg-Cu alloys have become one of the important lightweight structural materials in aerospace due to their excellent properties such as low density, high specific strength and high specific stiffness [1,2,3,4,5,6]. The initial design requirements for ultra-high-strength aluminum alloys were high strength and low toughness. Based on this, corrosion resistance requirements were later introduced and gradually developed towards corrosion resistance and ultra-high-strength toughness [7]. Material composition optimization, large plastic deformation, and exploring heat treatment regimes are three effective methods of improving the corrosion resistance and toughness of AL-Zn-Mg-Cu alloys.

Zn is the main reinforcing element in Al-Zn-Mg-Cu alloys, and after heat treatment, it forms MgZn_2_ (η′ phase) reinforcing phase, which is usually co-lattice or semi-co-lattice with the matrix, and plays a major role in enhancing the comprehensive properties of Al-Zn-Mg-Cu alloys [8]. Researchers have increased the Zn content to 9.0–10.0% with a view to precipitating a large number of nanoscale MgZn phases (η′ phases) in the matrix, thereby improving the mechanical properties of the alloys. However, in the actual casting process, with the increase in Zn content, a large number of coarse Al-Cu-Mg-Zn tetragonal phases were also formed in the matrix in addition to the MgZn phase. In service, this insoluble phase leads to localized stress concentration and initiates cracks, which significantly reduces the mechanical properties of the component [9]. At the same time, the insoluble phase absorbs a large number of Zn and Mg elements, which reduces the content of the aging precipitation phase (η′ phase), and ultimately results in the mechanical properties of the components are difficult to meet the design requirements. Therefore, solving the problems of incipient second-phase organization, difficult control of macro and micro defects, and serious macro segregation of high-alloyed multi-component aluminum alloys during casting is the key to improve the properties of Al-Zn-Mg-Cu alloys.

Compared to conventional plastic deformation, a significant increase in strain is the main feature of the macroplastic deformation process, which introduces more tissue defects through large deformation in order to obtain sub-micron or even micron-sized ultrafine grains. The large plastic deformation (SPD) process, as a method that can effectively improve the material organization, refine the grain size, and enhance the mechanical properties [10], the large number of fine crystals and high dislocation density were obtained by applying a plastic strain greater than 2. Bakhshi et al. [11] obtained a large number of fine crystals and high dislocation density by applying a large plastic deformation and natural aging to 7005 aluminum alloy. The grain size was reduced to about 1 μm, and the yield strength of the alloy reached 400 MPa or more. LY12 aluminum alloy was subjected to 10 passes of a repeated upsetting and extrusion (RUE) process; the approximate cumulative true strain reached 8.926, and an equiaxed ultrafine organization with an average grain size of about 200 nm was obtained, and the yield strength reached 513.6 MPa [12]. In addition to refining the organization, large plastic deformation can also promote the fragmentation of the insoluble second phase. The fragmented second phase can improve the work hardening effect by limiting the dislocation motion [2,13,14,15]. Therefore, in order to avoid the shortcomings of high alloying aluminum alloy ingot deformation which is very prone to cracks, poor deformation strengthening effect, and high scrap rate, the large plastic deformation process has become an important means of enhancing the high strength and toughness Al-Zn-Mg-Cu alloys.

For high-alloyed Al-Zn-Mg-Cu alloys, it is difficult to achieve the high-performance service requirements of high-end equipment only through a single heat treatment. Many scholars have proposed to add pre-treatment means such as deformation and aging in the conventional heat treatment process, so as to achieve the goal of enhancing the subsequent aging precipitation effect and improving the mechanical properties of the new high Zn content Al-Zn-Mg-Cu alloys. Deschamps et al. [2] argued that an increase in the amount of pre-stretching would lead to a significant decrease in the peak strength. Dislocations induced a large precipitated phase size gradient in Al-Zn-Mg-Cu alloys, i.e., the size of precipitates away from the dislocations remained unchanged, and there was a large amount of precipitation near and on the dislocations. Han’s study [13] found that after pre-stretching by 2.3%, under all aging conditions, the precipitated particles within the grains became rough, and the density of precipitated phases at grain boundaries decreased that reduced the strength gap between the matrix and grain boundaries, thus increasing the fracture toughness of the alloy. However, after pre-stretching, coarse rod-like η precipitates are formed, leading to a reduction in strength. In contrast, some researchers have found that pre-stretching can improve the mechanical properties of Al-Zn-Mg-Cu alloys. Han Baoshuai et al. [14] found that the tensile strength of Al-Zn-Mg-Cu alloy could reach 813 MPa with 10.10% elongation after 3% pre-stretching as well as aging at 80 °C/12 h and 120 °C/8 h. The tensile strength of Al-Zn-Mg-Cu alloy could be increased by 3% pre-stretching. However, when the amount of pre-stretching is increased to 4%, it will cause coarsening of the precipitated phase and reduces its strength. Zou et al. [15] systematically investigated the different effects of pre-stretching on the mechanical properties and microstructure of five Al-Zn-Mg-Cu alloys with different Zn/Mg ratios. The results showed that pre-stretching leads to a significant increase in the hardness of the Al-Zn-Mg-Cu alloy with the highest Zn/Mg ratio, and the maximum strengthening effect occurs because the precipitated phase of the alloy becomes denser and finer under 2% pre-stretching. On the contrary, pre-stretching leads to a decrease in the hardness of the lowest Zn/Mg ratio alloys (T′-phase strengthened) due to the accelerated coarsening of the precipitated phase.

In this paper, different pre-deformation treatments are carried out for Al-Zn-Mg-Cu alloy components prepared by reciprocal upsetting + counter-extrusion deformation process. The effects of pre-deformation on the aging precipitation phase and the optimal deformation heat treatment scheme are investigated, and the mechanism of toughening and the kinetics of the second phase precipitation are analyzed, which provides a new idea for the preparation of 7xxx-series aluminum alloys, further enriches the toughening means of 7xxx-series aluminum alloys, and gives theoretical support for the engineering application of the new type of Al-10.0Zn-3.0Mg-2.8Cu alloy.

## 2. Materials and Methods

**Figure 1:**
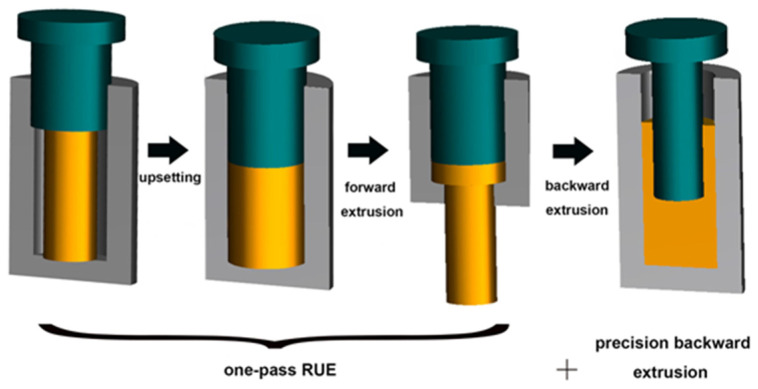
Schematic diagram of deformation process.

In this study, a new type of Al-Zn-Mg-Cu alloy developed by Central South University was used and homogenized at 470 °C × 20 h, and one reciprocal upsetting + counter-extrusion test was used to prepare Al-Zn-Mg-Cu components (as shown in Figure 1). The procedure is as follows: Firstly, an initial billet with dimensions Φ250 mm × 310 mm is obtained by cutting wires. The blank is preheated at 470 °C for 3 h. The preheating temperature of the mold was 20 °C higher than that of the billet, and the preheating time was 3.5 h. The extrusion preform experiments were carried out in a 3000-ton hydraulic press, as shown in Figure 1, and the billet was upset to change its diameter from 250 mm to 340 mm. The billet obtained in the previous step was then extruded to change its diameter from 340 mm to 180 mm. The preform was held at 430 °C for 2.5 h prior to the backward extrusion, with the die preheated to 20 °C higher than the billet preheating temperature. The preheating temperature of the mold was 20 °C higher than the preheating temperature of the blank, and the holding time was 3.5 h. After insertion of the held billet into the die, a backward extrusion test was carried out with a 3000-ton hydraulic press. The chemical compositions of the materials are shown in Table 1.

In order to minimize the occurrence of natural aging of the specimens during the experimental process, the pre-tensioned specimens were sampled prior to the solution treatment. The solution treatment was carried out in an air-circulating heating furnace, followed by water quenching at room temperature. The pre-stretching rate was set to 0.5 mm/min, and the deformation was selected as 0% (unstretched), 2%, 5%, and 7%, respectively. Artificial aging treatment was carried out in an air-circulating low-temperature oven at an aging temperature of 120 °C.

**Figure 2:**
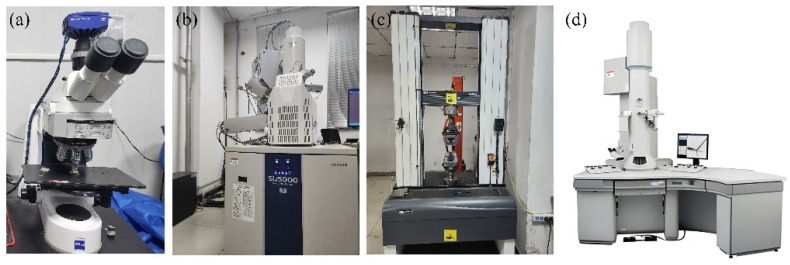
Instrument pictures (**a**) optical microscope (OM), (**b**) scanning electron microscope (SEM), (**c**) electronic tensile testing machine, (**d**) transmission electron microscope (TEM).

The microstructures of the specimens were characterized using an optical microscope (Zeiss A1m (Carl Zeiss AG, Oberkochen, German)) (Figure 2a), scanning electron microscope (SEM, Hitachi SU5000 (Hitachi Limited, Tokyo, Japan)) (Figure 2b) and transmission electron microscope (TEM, FEI Tecnai G20, (FEI Company, Hillsboro, OR, USA)) (Figure 2d). The OM and SEM specimens were mechanically abraded and polished, and then the corrosive agent was configured in accordance with 1 mL of HF + 1.5 mL of HCl + 2.5 mL of HCl + 2.5 mL of HNO_3_ + 95 mL of H_2_O, and the corrosion time was 10–20 s. The volume fraction of the precipitated phase was counted using SEM. The thinning parameters were: high-pressure (6.3 kv), large-angle (4.5°) thinning for 35 min, then low-pressure (5.5 kv), small-angle (2.0°) thinning for 15 min.

In this paper, the mechanical properties are tested by tensile performance test. The flaky tensile specimen was used with a gauge length of 25 mm. The tensile test was carried out on the Instron3382 (Instron, Boston, MA, USA) electronic tensile testing machine at a tensile rate of 1 mm/min (Figure 2c). The yield strength and tensile strength of different samples were obtained by extensometer, and three samples were tested under the same conditions.

## 3. Results and Analysis

### 3.1. Initial Microstructure Performance Analysis

**Figure 3:**
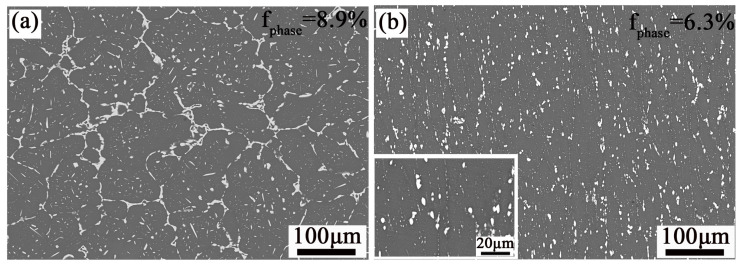
SEM images: (**a**) homogenized state; (**b**) extruded state.

Figure 3 shows the microstructure and second-phase distribution of Al-10.0Zn-3.0Mg-2.8Cu alloy in homogenized and extruded states. The organization after homogenization consists of coarse grains, and the grain morphology after deformation is elongated along the extrusion direction. It can be seen that there are mainly two types of second phases in the matrix. One type is the granular phase uniformly dispersed inside the α(Al) grains. The other type is the coarse insoluble phase present at the grain boundaries. The area fraction statistics of the second phase are shown in the upper right corner of the image(f_phase_). The area fraction of the second phase in the homogenized state specimen is 8.9%, in which the coarse grain boundary insoluble phase dominates. The SEM image of the formed member shows that in Figure 3b, the second phase distributed at the grain boundaries undergoes significant fragmentation, and a large number of fine dispersed second phase particles appear inside the grains. At this point, the area fraction of the second phase of the alloy is reduced by 2.6% compared to the homogenized state. This indicates that the cyclic upsetting and extrusion process promotes the fragmentation of the coarse second phase and the precipitation of the fine phase inside the grain.

**Figure 4:**
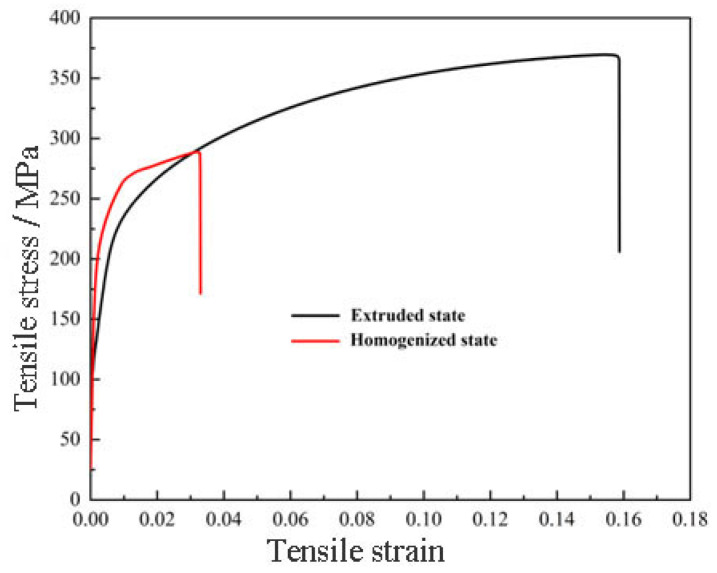
Room temperature mechanical properties of homogenized and extruded states.

Figure 4 shows the tensile stress-strain curves of Al-Zn-Mg-Cu alloy in homogenized state and extruded state. The strength and plasticity of Al-Zn-Mg-Cu alloy are improved after reciprocal upsetting + precise counter-extrusion deformation, and its tensile strength is increased to 369.5 MPa, which is 25.3% higher than the performance of the alloy in the homogenized state; the elongation of the Al-Zn-Mg-Cu alloy tailstock is 14.6%, which is 342.4% higher than that of the homogenized state. It can be seen that the new forming process ameliorates the mechanical properties of the alloys, but the strength of the tail frame in the extruded state is still relatively low, which makes it difficult to meet the high-strength service requirements. Therefore, it is necessary to control the distribution of the second phase and the grain organization of the alloy through pre-deformation and heat treatment for the sake of improving the comprehensive performance of the alloy and realize the toughening of the alloy.

### 3.2. Effect of Pre-Deformation on the Peak Age-Hardening Behavior of Alloys

**Figure 5:**
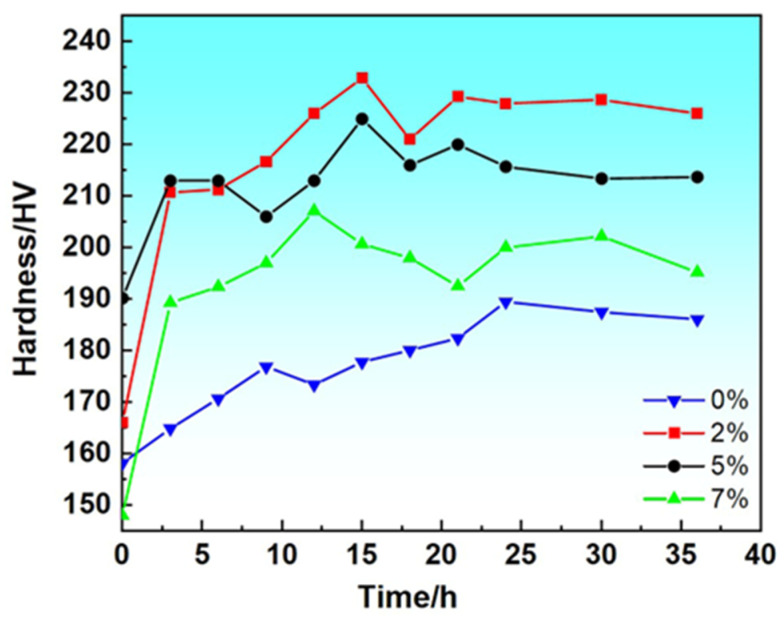
Age-hardening curves of alloys treated at 470 °C/3 h + pre-stretching (0%, 2%, 5%, 7%).

After the specimens were solution treated and quenched, the specimens were pre-stretched and deformed by 0%, 2%, 5%, and 7%, respectively at room temperature, and then artificially aged at 120 °C. The hardness changes are shown in Figure 5. As can be seen from the figure, the hardness value of the alloy increased rapidly within 0–3 h, and then the growth rate gradually slowed down. It can be seen that the sample with 0% pre-distortion has the lowest peak hardness, while the sample with 2% pre-distortion has the largest peak hardness value, and the peak hardness of the sample decreases with the increase of pre-distortion. As the amount of pre-deformation increases, the time required for the alloy to reach peak aging decreases from 24 h under no pre-deformation to 12 h after 7% pre-deformation, indicating that the pre-stretching deformation not only improves the hardness of the aging organization of Al-Zn-Mg-Cu alloy but also effectively reduces the time required for the alloy to reach the peak aging hardness.

### 3.3. Effect of Pre-Deformation on the Microstructure of Alloys Subjected to Peak Aging

**Figure 6:**
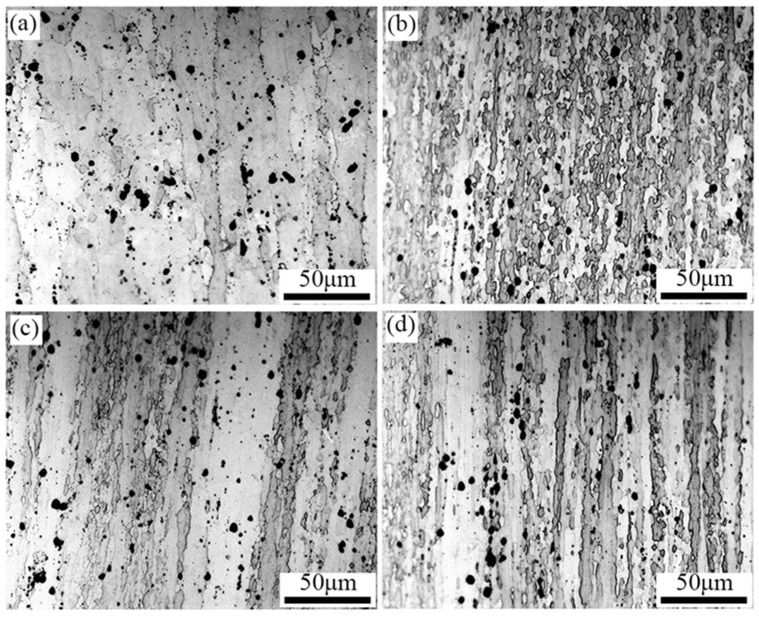
OM diagram of alloy after treatment at 475 °C/3 h + pre-stretching+ aging. (**a**) 0%, (**b**) 2%, (**c**) 5%, (**d**) 7%.

As shown in Figure 6, the metallographic morphology of the alloy after peak aging with pre-deformations of 0%, 2%, 5%, and 7%, respectively. When no pre-distortion is introduced, the alloy is mainly composed of coarse elongated grains (about 18.34 μm in width) and a small number of equiaxed grains.

**Figure 7:**
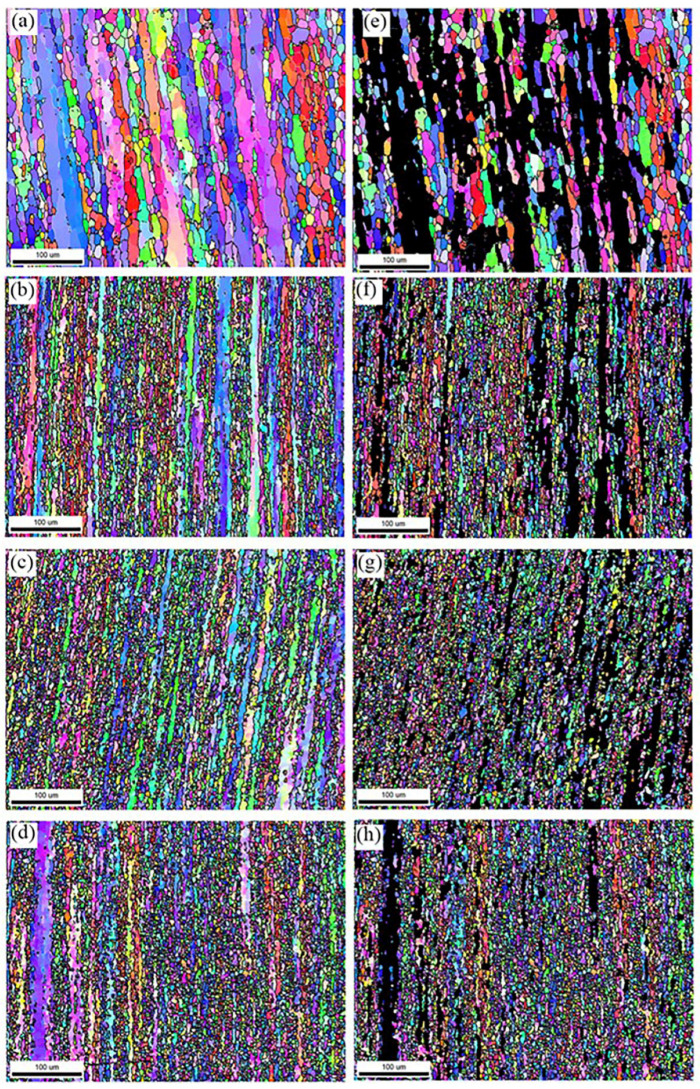
EBSD and recrystallized grain diagrams of alloys after treatment at 475 °C/3 h + pre-stretching + aging. (**a**,**e**) 0%, (**b**,**f**) 2%, (**c**,**g**) 5%, (**d**,**h**) 7%.

In order to further investigate the influence law of deformation and heat treatment on the grain organization, the peak aging EBSD structure of the alloy under different pre-stretching amounts was analyzed, and the EBSD diagram and recrystallization structure diagram are shown in Figure 7. From the analysis in Figure 7a, it can be seen that the peak aging alloy structure without the introduction of pre-stretching has elongated grains along the axial direction of the cylinder member, most of the metamorphic tissues are retained in the matrix, and few regions of recrystallization occur. From the analysis in Figure 7b–d, it can be seen that the elongated metamorphic tissues in the peak aging tissues after the introduction of pre-deformation are reduced in a large number, indicating that recrystallization occurs in the process of deformation heat treatment. Under the strain accumulated in the pre-deformation, LAGBs are gradually transformed into HAGBs, and a large number of fine isometric crystals along the vicinity of the original grains are seen in peak aging alloys, as seen in the figure. After 2% pre-deformation treatment, the elongated metamorphic tissue in the organization was reduced in large quantities, which indicated that recrystallization occurred during the deformation heat treatment process. Under the strain accumulated in the pre-deformation, the LAGBs gradually changed into HAGBs, and a large number of fine equiaxial crystals appeared along the vicinity of the original grains in the peak-ageing-state alloy, as can be seen in the figure. Continuing to increase the degree of pre-deformation to 5% and 7%, the grain structure is comparable to the 2% pre-deformation structure.

The recrystallized volume fraction and average size of recrystallized under different deformation heat treatment regimes are statistically presented in Table 2. The recrystallized volume fraction increases with the increase in pre-deformation, and when the pre-stretching amount is 5%, the recrystallized grain size is the smallest, which is 4.6 μm. When the pre-stretching amount is 7%, the recrystallized volume fraction reaches 82.8% and the size of recrystallized grain increases to 7.4 μm.

### 3.4. Effect of Pretreatment on Precipitated Phases of Alloys Subjected to Peak Aging

**Figure 8:**
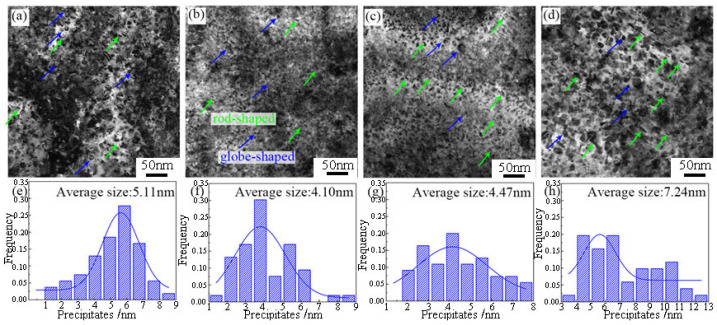
TEM image and precipitation phase size statistics of the alloy after 475 °C/3 h + pre-stretching (0%, 2%, 5%, 7%) + aging treatment (**a**,**e**) 0%; (**b**,**f**) 2%; (**c**,**g**) 5%; (**d**,**h**) 7%.

Figure 8 demonstrates the TEM morphology of the alloy in the peak aging state after different pre-deformation treatments, and the statistical plots at the bottom of the figure show the size statistics of the precipitated phases in the corresponding states. Figure 7a shows the microstructure of the 0% specimen, and it can be observed that the intracrystalline tissue is distributed with rod-like and spherical precipitated phases, mainly η′ and η phases, with an average size of 5.11 nm. The size of the precipitated phases in the 2% tissue decreases slightly, with an average size of 4.10 nm, which is attributed to the fact that dislocations introduced by the pre-distortion can generate more nucleation sites. This promotes the formation of the dispersed η′ phase during the subsequent aging process. On the other hand, the precipitates in 5% samples are coarsened and spheroidized, with an average size of 4.47 nm. The precipitates of 7% are larger and mainly spherical, with an average size of 7.24 nm. This is due to the fact that more defects, such as dislocations, are introduced with the increase of pre-deformation, which accelerates the precipitation of the precipitated phase during the artificial aging after the deformation, and the precipitated phase is more prone to nucleation and becomes larger in size. The decrease in the hindering effect of dislocations in the coarse precipitated phase leads to the weakening of the contributing effect to the strength, resulting in a decrease in strength.

### 3.5. Effect of Different Deformation Heat Treatment Regimes on the Peak Aging Tensile Mechanical Properties of Alloys

**Figure 9:**
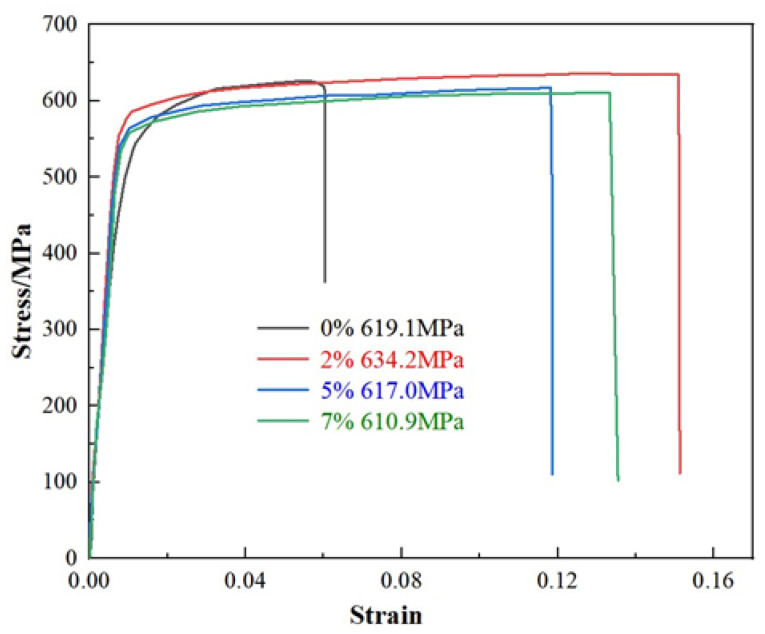
Room temperature tensile properties of Al-10.0Zn-3.0Mg-2.8Cu with different pre-deformations (0% 619.1 MP, 2% 634.2 MP, 5% 617.0 MP, 7% 610.9 MP).

The tensile property curves of the alloys after different pre-deformation treatments are given in Figure 9, and the corresponding specific mechanical property statistics are given in Table 3. From the statistics in Table 3, it is clear that the introduction of pre-deformation can significantly increase the elongation of the samples compared to the elongation of the samples without pre-deformation in both cases.

At a pre-stretching amount of 2%, the strong plasticity of the samples was optimal compared to the others, which corresponded to the refined and uniform grain structure and diffuse and dense precipitation structure. The tensile strength of the samples decreased to 617.0 MPa and 610.9 MPa when the pre-stretching amounts were 5% and 7%, respectively, which was attributed to the coarsening of the precipitated phases leading to the reduction of mechanical properties.

In summary, the mechanical properties of the alloy can be effectively enhanced by adding pre-distortion after solid solution. When the pre-stretching amount is 2%, the strength and toughness of the peak aging sample reach their best values. When the pre-stretching amount is greater than 2%, the precipitated phase starts to coarsen, resulting in the reduction of properties.

## 4. Discussion

### 4.1. Mechanism of Pre-Deformation Effect on Precipitation Behavior

**Figure 10:**
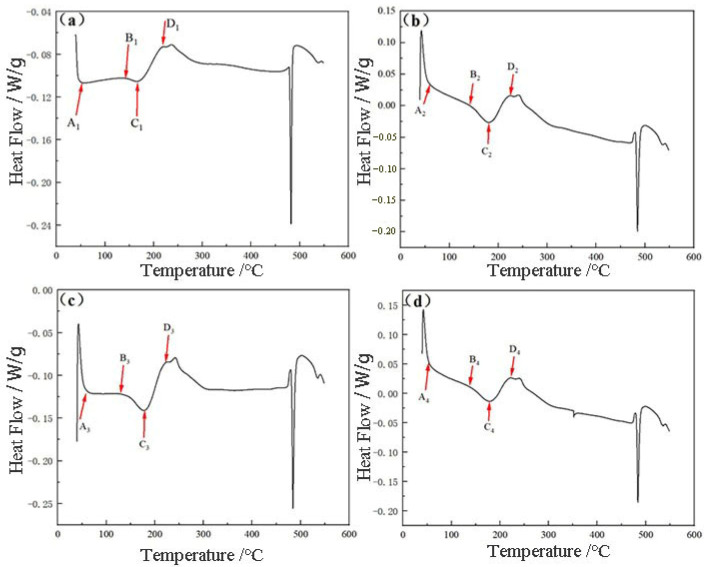
DSC curves for different deformation heat treatment conditions. (**a**) 0%, (**b**) 2%, (**c**) 5%, (**d**) 7%.

In order to investigate the precipitation behavior of the alloy under different deformation heat treatments, DSC experiments were carried out, as shown in Figure 10. It can be observed that there are two typical heat-absorbing peaks—A and C—on the curve, indicating the dissolution of some phases. Meanwhile, the other inverse peaks accompanying the heat absorption peaks are exothermic peaks—labeled as B and D—indicating the precipitation of a phase. According to the previous study [8], the formation of the inspiral peak A corresponds to the dissolution of the GP (Guinier–Preston) region. The exothermic peaks of B and D represent the precipitation of the η′ and η phases, respectively, and C represents the back dissolution of the η′-enhanced phase. With the increase in temperature in Figure 10a, at peak B1, the reinforced phase η′ of Al-Zn-Mg-Cu alloy precipitates along the GP zone at about 56.7 °C, which is the main precipitated phase in the peak aging stage. After a longer aging treatment, the η phase precipitates after η′ starts to dissolve back at peak C1. It can be seen that the intensity of the dissolution peak in the GP zone increases in the pre-stretched samples with deformations of 2%, 5%, and 7% compared to the un-pre-stretched samples, and the intensity of the precipitation peak of the η′ phase is higher than that of the un-pre-stretched samples, which implies that the volume fractions of the GP zone and the η′ phase in the peak aging samples of the pre-stretched samples are more than those of the un-pre-stretched samples. It is worth noting that the temperature of the dissolution peak (146.4 °C) corresponding to the dissolution of the η′ phase of the 7% sample in Figure 10d is significantly shifted toward higher temperatures, suggesting that the η′ phase of the artificially aged tissues has a larger size at 7% pre-stretching, and higher temperatures are required for dissolution. In addition, the areas of the precipitation and coarsening peaks of the η phase in the 7% sample are significantly larger than those in the 2% and 5% samples. This is due to the presence of a large number of dislocations as the non-uniform nucleation points of the η phase, which is rapidly precipitated and undergoes significant coarsening. Therefore, and thus the areas of the exothermic and heat-absorbing peaks corresponding to the precipitation of the η phase are larger in the sample with a pre-stretching of 7%. It is also observed that the pre-stretching has no effect on the order of precipitation of the reinforced phase in the figure.

Numerous researchers [16,17,18,19,20,21,22] have studied the precipitation kinetics and yield strength calculation models for aluminum alloys during aging in some depth, and after generalizing and fitting the experimental data, Luiggi et al. [23] obtained an empirical equation for the precipitation kinetics of the organization (JMAK equation). In this section, the JMAK equation is used to calculate the volume fraction and precipitation rate of the second phase precipitated from Al-Zn-Mg-Cu alloy during aging and to calculate the precipitation activation energy and constant of the second phase.

Hardness measurements were used to study the precipitation kinetics of the second phase by considering the hardness values from the start of aging (HSA) to the peak aging hardness (HP). Thus, Equation (1) can be used to calculate the volume fraction *f* from the hardness (H) values, and the phase transition can then be investigated using the JMAK model shown in Equation (2), which has been successful in calculating the activation energy of the phase transition for different alloys, Q, based on Equation (3):

(1) $$f=\left(H-H_{SA}\right)/\left(H_{P}-H_{SA}\right)$$

(2) $$f=1-exp\left(-{\left(kt\right)}^{n}\right)$$

(3) $$k=k_{0}exp\left(-Q/RT\right)$$

The implicit function containing the volume fraction of the precipitated phase is:

(4) $$F=n\left(1-f\right){\left[-\ln\left(1-f\right)\right]}^{\frac{n-1}{n}}$$

It can be found that the kinetic equation for tissue precipitation is:

(5) $$\ln\left[\frac{df}{dT}\frac{\frac{dT}{dt}}{n\left(1-f\right){\left[-\ln\left(1-f\right)\right]}^{\frac{n-1}{n}}}\right]=\ln k_{0}-\frac{Q}{RT}$$

where dT/dt is the heating rate during isothermal aging.

Before calculating the kinetic equations for the GP zone, η′ phase, and η phase under different deformation heat treatment conditions, the value of *n* must be discussed. The relationship between the phase transformation mechanism of the alloy and the value of n is given in Table 4, which provides a reference for the selection of *n*. From Table 4, it can be seen that the value of *n* increases with the increase of the precipitation rate. According to the JMAK equation [23] in the transformation mechanism of long-range diffusion-controlled growth, the phase transition constants of the GP region and η′ and η phases are selected as *n* = 2/3, *n* = 1, and *n* = 1, respectively.

**Figure 11:**
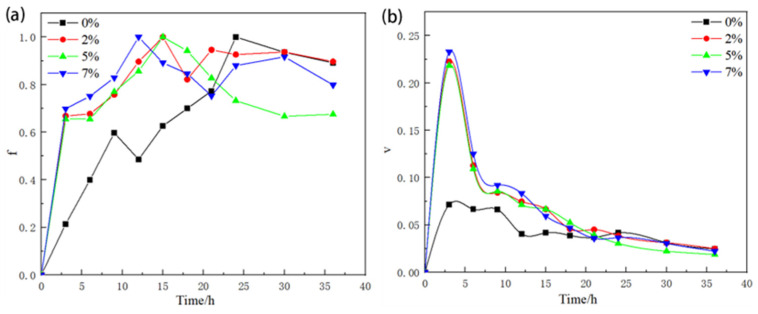
Second-phase precipitation kinetics: (**a**) variation in volume fraction with time of efficacy for different amounts of pre-deformation; (**b**) variation of precipitation velocity with time of efficacy for different pre-deformation amounts.

Taking the unstretched DSC curve Figure 10a as an example, it can be analyzed that the formation temperature range interval of the GP zone is 53.982 °C to 86.09 °C, the formation temperature range interval of the η′ phase is 153.023 °C to 176.763 °C, and that of the η phase is 198.033 °C to 226.147 °C. The overlapping peaks were separated, and the precipitation rate was determined by Equation (3), which is the same as that of the second phase after 475 °C+3 h solid solution quenching treatment. After the separation of the overlapping peaks, the precipitation volume fraction and precipitation rate of the second phase after 475 °C + 3 h solid solution quenching treatment can be obtained according to the separation results and Equation (3), and the results are shown in Figure 10. During the aging process, the volume fraction of the precipitated phase will fluctuate with the changes in the size and quantity of the second phase. In Figure 11a, the volume fraction of the precipitated phase gradually increases as the amount of pre-deformation increases, but when the amount of deformation reaches 7%, this adversely affects the properties of the alloy. During the aging process, due to the increase in dislocations introduced under 7% pre-deformation conditions, it provides a channel for the precipitation of solute atoms, which results in the fastest precipitation of the second phase after the same aging time, as shown in Figure 11b.

**Figure 12:**
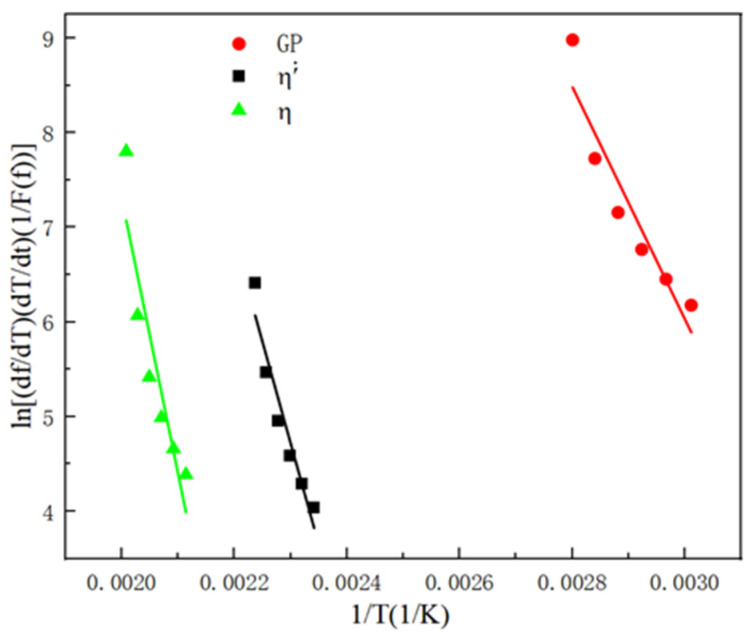
Determine the activation energy.

In Equation (5), n needs to be chosen according to the nucleation and growth mechanism. After that, the expression of F was derived to make a plot of ln[(df/dt)(v/F)] versus 1/T, whose slopes are the activation energies of precipitation in the GP region and η′ and η phases, as shown in Figure 12. Based on the slopes of Figure 12 and Equation (5), the kinetic parameters of precipitation in the GP region and η′ and η′ phases are derived as shown in Table 5, with the activation energies of the precipitation in the GP region and η′ and η phases of 102.059 kJ·mol^−1^, 178.147 kJ·mol^−1^, and 241.622 kJ·mol^−1^, respectively. The constants k0 are 4.14 × 1018 s^−^^1^, 2.87 × 1023 s^−^^1^, and 2.64 × 1028 s^−^^1^, respectively.

### 4.2. Strengthening Mechanism of Pre-Deformed Al-Zn-Mg-Cu Alloys

Precipitation strengthening is the main strengthening mode of Al-Zn-Mg-Cu alloy, and its high strength and toughness mainly depend on the type, quantity, size, volume fraction, and distribution of intracrystalline and grain boundary precipitation phases (GP zone, η′ phase, η phase) after aging, etc. GP zone, η′ phase, and η phase are the main strengthening phases of Al-Zn-Mg-Cu alloy.

The precipitation strengthening mechanism is mainly categorized into dislocation cut-through mechanism and dislocation bypassing mechanism. The critical size of the dislocation cut-through mechanism and bypassing mechanism is about 2 nm [24]. The TEM statistical results are displayed in Figure 10, which shows that the average size distribution of the precipitated phase shows a normal distribution, and the average size of the particles in all states is larger than the critical value, so the Orowan dislocation bypassing mechanism is used to explain the reinforcement mechanism [25,26].

The expression is as follows [7]:

(6) $${\Delta \sigma }_{\mathrm{or}}\approx {\beta f}^{1/2}r^{-1}$$

where β is a constant. f and diameter are the precipitated phase volume fraction and radius. It can be seen that the volume fraction of the precipitated phase is proportional to the strength, and the size of the precipitated phase is inversely proportional to the strength. The average size statistics of the precipitated phase under different pre-deformation conditions are shown in Table 6 below:

Combined with the DSC curves under different pre-deformation conditions in Figure 10, it can be seen that the intensity of the dissolution peaks in the GP region increases in the pre-stretched samples with deformation amounts of 2%, 5%, and 7% compared with to the un-pre-stretched samples, and the intensity of the precipitation peaks of the η′ phases is higher than that of the un-pre-stretched samples, which means that the volume fractions of the GP region and the η′ phases in the pre-stretched peak-ageing samples are more than that of the un-pre-stretched samples. The area of the dissolution peak of η′ phase also increases with the increase of deformation, which suggests that many fine η′ phases are mainly formed when peak aging is reached, and these η′ phases are unstable and undergo dissolution during the DSC warming process. It is noteworthy that the temperature of the dissolution peak corresponding to the dissolution of the η′ phase of the 7% sample is significantly shifted to the high temperature direction, which indicates that the η′ phase of the artificially aged tissue is larger in size at 7% pre-stretching, and a higher temperature is required for its dissolution, which is in agreement with the results demonstrated in the previous TEM images. Moreover, the areas of the precipitation and coarsening peaks of the η-phase in the 7% sample are significantly larger than those in the 2% and 5% samples. This is due to the presence of a large number of dislocations as the non-uniform nucleation points of the η-phase, which leads to rapid precipitation of the η-phase and significant coarsening of the η-phase. Therefore, the areas of the exothermic and heat-absorbing peaks corresponding to η-phase precipitation of the η-phase precipitation are larger in the pre-stretched sample.

In conclusion, according to the dimensions of the precipitated phases and the DSC curves in Table 6, it can be concluded that the average size of the precipitated phases is the smallest, and the volume fraction reaches a high level when the pre-deformation is 2%. According to Equation (6), it can be analyzed that the precipitated phases in the structure of 2% specimens contribute the most to the strengthening of the alloy, which corresponds to the value of the tensile mechanical properties.

**Figure 13:**
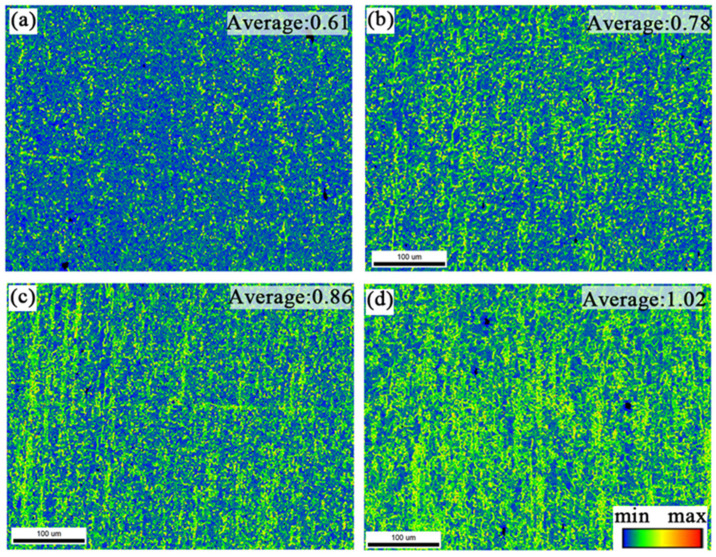
KAM plot of Al-Zn-Mg-Cu alloy tailstock. (**a**) pa0%; (**b**) pa2%; (**c**) pa5%; (**d**) pa7%.

**Figure 14:**
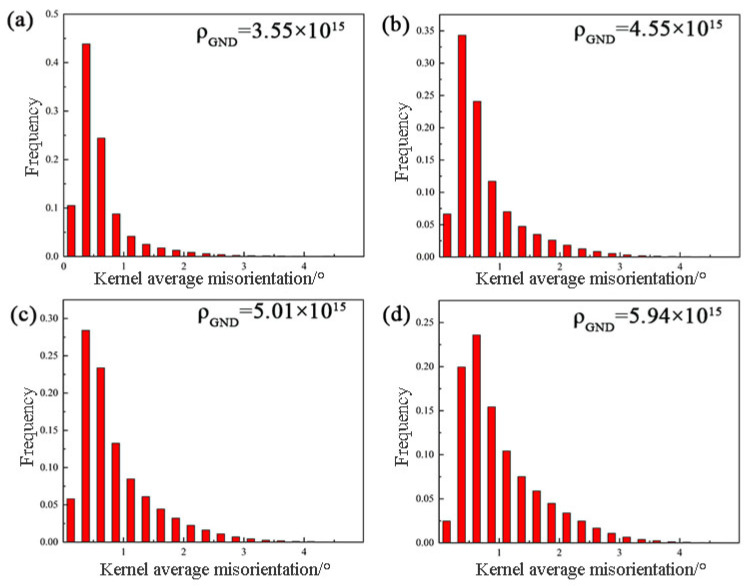
KAM histogram of Al-Zn-Mg-Cu alloy tailframe. (**a**) pa0%; (**b**) pa2%; (**c**) pa5%; (**d**) pa7%.

In order to investigate the effect of different pre-deformation amounts on the peak aging dislocation density, Figure 13 and Figure 14 demonstrate the average grain orientation difference plots and the statistical histograms of KAM values of the alloys after different pre-deformation treatments. At 0% to 7% pre-deformation, the local orientation difference of the peak aging state samples is significantly higher, and its average KAM value increases from 0.61 to 1.02. According to the histogram of KAM distribution, it can be seen that the KAM values have higher peaks at smaller angles, and with the increase of pre-deformation, the KAM values are more distributed at smaller angles. Theoretically, the local orientation difference angle and geometrically necessary dislocation density (ρ_GND) can be calculated using Equation (7) [7,27], and the EBSD scanning step value for all the specimens 1.2 μm.

(7) ρ=2θ/(μb)

The calculated geometrically necessary dislocation densities of the specimens under different pre-deformation conditions are shown in the histogram of the KAM distribution in Figure 14. As the amount of pre-deformation rises from 0% to 7%, which shows that the geometrically necessary dislocation densities of the samples in the peak aging state increase as the amount of pre-deformation rises from 0% to 7%, implying that the total dislocation densities within the alloys increase. This is partly due to the increase in the amount of pre-deformation, which introduces a large number of dislocations and increases the dislocation density, which is—on one hand—due to the fact that the increase of the amount of pre-deformation introduces a large number of dislocations that increase the dislocation densities., On the other hand, this is due to the introduction of a large number of dislocations by the increase of pre-deformation, which increases the dislocation density, and on the other hand, it is due to the pinning effect of the fine precipitates relative to the dislocations in the aging process, which inhibits the dislocations’ movement and increases the dislocation density.

The quantitative calculation of dislocation density allows the magnitude of its contribution to the strength of the material [14]:

(8) $${\Delta \sigma }_{d}={M\alpha \mathrm{Gb}\rho }^{1/2}$$

where M is the mean orientation factor (M = 3), α is a constant, G is the shear modulus (G = 26.1 GPa), b is the Parker vector (=0.286 nm), and ρ is the dislocation density. It can be seen that the alloy strength is proportional to the dislocation density. Thus, the contribution of dislocation strengthening to the strength of alloys in different deformed heat-treated states increases with the amount of pre-deformation. These dislocations not only provide channels for the precipitation of solute atoms but also increase the strength of the alloy. When the amount of deformation is increased to a certain extent, the dislocations inside the alloy are entangled and piled up with each other, and they impede the dislocation slip, so the strength of the alloy increases.

### 4.3. Fracture Mechanism of Pre-Deformed Al-Zn-Mg-Cu Alloys

**Figure 15:**
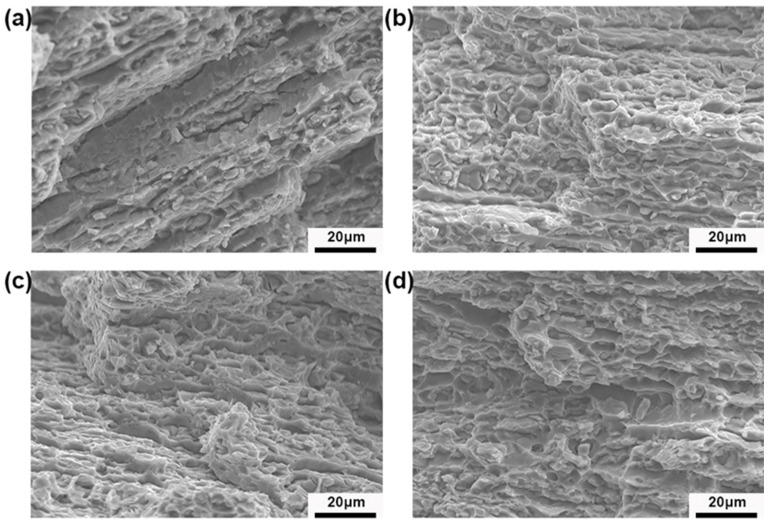
Fracture morphology of Al-Zn-Mg-Cu alloy tailstock. (**a**) pa0%; (**b**) pa2%; (**c**) pa5%; (**d**) pa7%.

Figure 15 shows the fracture morphology of the alloys after different pre-deformation treatments. As can be seen from the figure, under 0% pre-stretching conditions, the fracture surface is dominated by the deconstructed structure, the distribution of tough nests is more limited, and the fracture is a brittle fracture. After 2% pre-stretching, the fracture surface begins to be dominated by denser ligamentous fossa structures, and most of them are small ligamentous fossa structures. The small and dense toughness nests are the main reason for the increase in toughness of the alloy after 2% pre-stretch deformation. However, after 7% pre-deformation treatment, a relatively coarse second phase with concentrated distribution is formed in the matrix. Iin the plastic deformation process, the coarse second phase will become an obstacle in the process of dislocation slip, and a local stress concentration is then formed, which ultimately leads to the nucleation and growth of holes and microcracks and growth as well as the formation of a larger size of the ligamentous fossa structure. In addition, the coarse second-phase particles in the plastic deformation process also affect the coordination of deformation within the grain and between the grains and through its pinning effect on the grain boundaries, thereby reducing the ability of the alloy to co-deform, exacerbating the nucleation and growth of local holes and microcracks.

## 5. Conclusions

(1) When the amount of pre-deformation is 2%, the matrix hardness value reaches its highest, and the peak aging time of the alloy is 15 h. Compared to the unpretreated specimens, the hardness value of the matrix is increased from 189.5 HB to 232.9 HB, and the grain size is refined from 31.8 μm to 12.4 μm. By using the pre-stretching process after solid solution treatment, when the amount of pre-deformation is increased from 0% to 2%, the size of the precipitated phase of matrix decreases from 5.11 μm to 4.1 μm. When the amount of pre-deformation increases from 2% to 7%, the matrix precipitate phase undergoes coarsening, and its size increases from 4.1 μm to 7.24 μm. The deformation heat treatment process of aluminum alloy tailstock was finally determined as follows: solid solution (475 °C/3 h) + 2% pre-stretching + aging treatment (120 °C/24 h). A, at this time, the comprehensive performance of the alloy reached its optimum vale, the tensile strength reached 634.2 MPa, the yield strength increased to 12.4 μm, and the deformation heat treatment process of the aluminum alloy tailstock was determined as follows; 634.2 MPa, yield strength was 571.0 MPa, and the elongation was 15.2%.
(2) By analyzing the DSC curves and activation energies of the alloys under different pre-deformation conditions, it was found that the activation energies of the phases are lower under higher-pre-deformation conditions, so the introduction of pre-deformation accelerates the precipitation of the second phase in the alloys. The lower activation energy of precipitation increases the nucleation sites, which further promotes the diffuse distribution of the second phase in the alloy. At the same time, the higher number of nucleation sites limits the coarsening of the precipitated phase.
(3) Pre-stretching can promote the precipitation of the precipitated phase during the aging process, increase the volume fraction of the precipitated phase, and achieve precipitation strengthening of the alloy. As the amount of pre-deformation increases (less than 5%), the volume fraction of the second phase increases, and the strengthening effect of the precipitated phase on the alloy increases. However, when the pre-deformation amount is 7%, although the volume fraction of the precipitated phase increases, the precipitated phase is coarsened, which reduces the strengthening effect on the alloy and adversely affects its mechanical properties. The contribution of dislocation strengthening to the strength of the alloys in different deformation heat-treated states, on the other hand, increases with the increase in the amount of pre-deformation. These dislocations not only provide channels for the precipitation of solute atoms but also increase the strength of the alloy when the amount of deformation is increased. To a certain extent, the dislocations inside the alloy are entangled and piled up with each other and impede the dislocation slip.
(4) After 2% pre-stretching, the fracture surface begins to be dominated by denser ligamentous fossa structures, and most of them are small ligamentous fossa structures at this time. Small and dense tough nests are the main reason for the increase in toughness of the alloy after 2% pre-stretch deformation. However, after 7% pre-deformation treatment, a relatively coarse second phase with concentrated distribution is formed in the matrix. During the plastic deformation process, the coarse second phase will become a an obstacle in the process of slip dislocation and then the formation a of local stress concentration, ultimately leading to the nucleation and growth of holes and microcracks, creating a formation of a larger size of the toughness of the structure of the nests, which makes so that the toughness of the alloy begins to decline.

## Figures and Tables

**Table 1:** Chemical composition of aluminum alloys (wt.%).

Mg	Cu	Zn	Zr	Fe	Al
2.5–3.0	2.6–3.0	9–11	0.15	0.06	Bal.

**Table 2:** Recrystallized volume fraction and average size of Al-Zn-Mg-Cu alloy tailframes under different deformation heat treatment regimes.

Number	Deformation Heat Treatment System	Average Size of Recrystallization/μm	Recrystallization Volume Fraction/%
1	Ps0%	15.9	42.6
2	Ps2%	5.9	65.8
3	Ps5%	4.6	66.6
4	Ps7%	7.4	82.8

**Table 3:** Room temperature mechanical properties of different states of Al-10.0Zn-3.0Mg-2.8Cu.

Condition	YS (Tensile Yield Strength) (MPa)	UTS (Ultimate Tensile Strength) (MPa)	EL (Elongation) (%)
0%	569.5	619.1	6.0
2%	571.0	634.2	15.2
5%	555.7	617.0	11.9
7%	551.3	610.9	12.2

**Table 4:** Phase transformation mechanism of alloys as a function of n value.

Conditional	*n*
Growth from small size increases nucleation rate	>2.5
Constant nucleation rate	2.5
Reduced nucleation rate	1.5–2.5
Zero nucleation rate	1.5
Sizable particle precipitation in the initial volume	1
Needle and plate precipitation of limited length size	1
Long cylinder precipitation roughening	1
Coarsening of large flake precipitation	0.5
Dislocation precipitation	2/3

**Table 5:** Kinetic parameters for the precipitation of the GP zone, η′ and η′ phases.

Phases	Peak Temperature/°C	Q (Hot Activation Energy)/(kJ·mol^−1^)	k_0_ (Constant)/s^−1^
GP	67.912	102.059	4.14 × 1018
η′	158.262	178.147	2.87 × 1023
η	215.003	241.622	2.64 × 1028

**Table 6:** Average dimensions of precipitated phases of Al-Zn-Mg-Cu alloy tailframes in different deformation heat treatment regimes.

Number	Deformation Heat Treatment System	Average Size of Precipitated Phase/nm
1	0%	5.11
2	2%	4.10
3	5%	4.47
4	7%	7.24

## Data Availability

Data is contained within the article.
